# University of the Witwatersrand physiotherapy undergraduate curriculum alignment to medical conditions of patients within Gauteng state health facilities

**DOI:** 10.4102/sajp.v73i1.362

**Published:** 2017-06-29

**Authors:** Mokgobadibe V. Ntsiea, Witness Mudzi, Nicolette Comley-White, Heleen Van Aswegen, Benita Olivier, Ronel Roos, Sonti Pilusa, Joanne Potterton, Hellen Myezwa, Natalie Benjamin, Vaneshveri Naidoo

**Affiliations:** 1 Department of Physiotherapy, School of Therapeutic Sciences, Faculty of Health Sciences, University of the Witwatersrand, South Africa

## Abstract

**Background:**

The healthcare sector requires graduates with the ability to confidently assess and manage the majority of the medical conditions seen in hospitals.

**Objective:**

To establish whether the most prevalent medical conditions treated by physiotherapists in Gauteng (South Africa) state health facilities align with the University of the Witwatersrand (Wits) physiotherapy curriculum.

**Methods:**

This was a retrospective review of condition-related statistics from physiotherapy departments within the Gauteng province state health facilities. Data from all Gauteng government hospitals that had submitted at least 75% of their physiotherapy condition–related statistics to the provincial statistics coordinator from January 2012 to December 2014 were considered and compared to medical conditions covered in the Wits 2015 physiotherapy curriculum to check if all conditions listed in the Gauteng statistics appeared within the Wits curriculum document. The number of teaching hours for the common conditions was noted to check the emphasis given to these conditions in the curriculum.

**Results:**

Eighty-three per cent of the hospitals submitted 75% of their monthly statistics. Overall, the most common conditions treated were lower limb fractures (13%) followed by stroke (7.6%) (*n* = 705 597). Within the neuro-musculoskeletal category, the most common conditions after lower limb fractures were soft tissue injuries (15.1%) (*n* = 330 511). The most common cardiopulmonary conditions were tuberculosis (24.9%), followed by pneumonia (13.8%) (*n* = 94 895). The most common neurological conditions were stroke (30.9%) followed by cerebral palsy (17%) (*n* = 174 024). Within the non-specified categories, the number of intensive care unit (ICU) patients was the highest (23%), followed by sputum induction (21%) (*n* = 138 187). The most common conditions that were emphasised within the Wits curriculum as indicated by the teaching hours: fractures, 14.5 (66%) of 22 third-year orthopaedics hours; stroke, 30 (73%) of 41 third-year neurology hours; soft tissue injuries, 18 (38%) of 48 fourth-year neuro-musculoskeletal hours; back lesions, 24 (50%) of 48 fourth-year neuro-musculoskeletal hours; and ICU patients, 30 (79%) of 38 fourth-year cardiopulmonary hours.

**Conclusion:**

The Wits physiotherapy curriculum covers all medical conditions treated by physiotherapists within the Gauteng state health facilities, and overall, the curriculum prepares the students to practise in a variety of situations.

## Introduction

The development of physiotherapy is dependent on the abilities and potential of the graduates, and these graduates need to have skills that are relevant to the needs of the health sector to enable them to practise competently and with confidence (Hunt et al. 1998). Many physiotherapy patients present with cardiopulmonary, neurological and musculoskeletal conditions, and thus academic programmes need curricula that facilitate student learning in preparation for these conditions; however, specific conditions to be covered should be appropriate to the current and future needs of their country’s health sector (Council of Canadian Physiotherapy Programmes 2009). Therefore, if our graduates cannot manage conditions seen in the South African (SA) health sector, they will not be relevant.

The healthcare sector requires physiotherapy graduates who can enter its employ with the ability to confidently assess and manage the majority of medical conditions seen in its hospitals. Universities aim to equip graduates with broad generic, transferable skills in preparation for a path of lifelong learning (Cowan, Norman & Coopmah 2005). Undergraduate training, especially curriculum content is thus instrumental in order to meet the needs of both the employer while ensuring lifelong learning for the graduates (Higgs, Higgs & Neubauer 1999). Therefore, to meet the needs of the healthcare sector, a curriculum is supposed to have current clinical skills as well as cover a wide variety of medical conditions that will be encountered during clinical practice (Brown, Brown & Roever 2006). Thus, review of the curriculum to meet the national needs is required for relevance of training of the health professionals (Mumbo & Kinaro 2015). Failure of the curriculum to keep pace with the national health conditions leads to the deterioration of the quality of graduates (Awases et al. 2013). However, in keeping pace with the national needs, all conditions and clinical skills must be fitted within the 480 credits of training over the four years of study.

As the content of the undergraduate curriculum expands to accommodate the changes in disease burden and the growth in knowledge, it should be remembered that the length of the course remains the same and thus the programme should not be overloaded (Chipchase, Williams & Robertson 2008). When adding additional medical conditions to the curriculum, it is important to consider evidence that supports management of these conditions. However, it has to be noted that there is no clear and accepted model for curriculum decision-making based on best available research evidence (Chipchase et al. 2008). Thus, in some instances, if there is evidence of benefit to the patient, albeit low-level evidence, some medical conditions may be included in the curriculum if they are common because training should meet the needs of local communities within their unique context (Pinnock & Jones 2008).

When developing or revising a curriculum, a good starting point would be the determination of the diagnoses or medical conditions that are appropriate to include or exclude in the undergraduate physiotherapy degree within a SA context in order to be responsive to the national disease burden. This is needed so that the universities can produce graduates who are well prepared for clinical work within SA while also being prepared to cope in a variety of clinical contexts outside of SA. This is supported by the Witwatersrand (Wits) physiotherapy department mission statement, which refers to graduating physiotherapists committed to meeting the health needs of all communities in SA, appropriately and cost effectively. Significant sociopolitical changes in the 1990s in SA prompted the Wits department of physiotherapy to begin the process of questioning the relevance and appropriateness of its undergraduate curriculum (Stewart et al. 1994; Wallner & Stewart 1994). This interrogation has been ongoing.

Each physiotherapy department within the Gauteng state hospitals is expected to submit monthly patient condition–specific statistics to a central provincial coordinator. Information gained from the patient statistics is used for health system monitoring and planning purposes such as motivation for posts and determining in-service training requirements (Health Metrics Network 2008). Physiotherapy training institutions can also use this information to check the relevance of the training programmes in addressing the most common medical conditions or injuries within our communities. Thus, information gained from the patient statistics can be used to inform curriculum content for training institutions. This information can also be used to check if the scope of the physiotherapy minimum training standard as set by the Health Professions Council of SA covers most of the medical conditions within the SA government hospitals. Ideally, data to make such decisions should be based on national patient statistics. However, each province has its own physiotherapy data capturing system, which differs from province to province, and thus it is difficult to make comparisons across provinces.

Relevant data for patient statistics may come from the national-level database of patients’ medical conditions that are captured through the SA District Health Information System (DHIS). However, most of the DHIS data are not physiotherapy specific. The DHIS does not capture neuro-musculoskeletal, neurological or orthopaedic conditions. In some instances, data that relate to physiotherapy are captured as mortality rates instead of morbidity such as in the study by Bradshaw et al. (2010) where the top six causes of death in SA are reported as HIV/AIDS, tuberculosis, pneumonia, diabetes, stroke and obstructive lung diseases. For the purposes of this study, DHIS data were not used because we needed morbidity data for all clinical areas of physiotherapy (musculoskeletal, neurological and orthopaedics).

Only data from Gauteng hospitals were considered in this study. Primarily because physiotherapy patient statistics are not collated nationally, and thus there is no standardised data collection sheet for all provinces. So, data from the Gauteng province were analysed as a starting point until such time that there is a standardised physiotherapy data collection sheet across provinces. Gauteng is the province of choice for this study as it has the largest SA population (South African Statistics 2012).

The aim of this study was to establish whether the most prevalent medical conditions treated by physiotherapists in Gauteng state health facilities were covered in the Wits physiotherapy curriculum.

## Methods

This was a retrospective review based on condition-related statistics collected from physiotherapy departments within the Gauteng province state health facilities. To review the Wits curriculum, we looked at the content of the curriculum to identify medical conditions covered for each clinical area. Data from all Gauteng government hospitals that submitted at least 75% (i.e. at least nine months per year) of their physiotherapy condition–related statistics to the provincial statistics coordinator from January 2012 to December 2014 were considered and compared to medical conditions covered within the Wits 2015 physiotherapy curriculum. Teaching timetables were also checked in case there were medical conditions that appeared on timetables that were not in the curriculum or course objectives. The amount of teaching time allocated to the most common conditions was noted from the physiotherapy teaching timetables.

A condition-related data capturing sheet was used to capture data for conditions treated by physiotherapists within the Gauteng government health facilities. The total number of patients seen during this time (2012–2014) was noted and the names of the health facilities were captured separately to ensure anonymity. Wits summary of the medical conditions was captured and organised into categories similar to those for the Gauteng health facility statistics (neuro-musculoskeletal, cardiopulmonary, neurology, including paediatrics for each category, other or non-specified categories).

A research assistant approached the chief physiotherapist who is responsible for collating physiotherapy statistics for Gauteng to acquire the required data. Three data collection researchers scanned the data for relevant information and captured the information on a spreadsheet, which was used to interpret results. Data from the hospitals were summarised using frequencies and percentages and university data were checked to establish if the most common hospital conditions appeared within the curriculum. The amount of time allocated for teaching the most common conditions was noted to check the emphasis of these conditions within the curriculum.

## Results

Data were received from the following Gauteng municipalities: City of Johannesburg metropolitan, City of Tshwane metropolitan, Ekurhuleni metropolitan, Sedibeng district, Emfuleni local, Lesedi local, Midvaal local, Metsweding district, Kungwini, Nokeng tsa Taemane, West Rand district, Merafong city local, Mogale city local, Randfontein local and Westonaria local.

Data were expected from 30 facilities. In 2012, 28 facilities (93%) submitted but only 21 (75%) of these facilities submitted more than 75% of their statistics (i.e. minimum of 9 months out of the expected 12 months). In 2013, 28 facilities (93%) submitted, but only 15 (54%) had more than 75%. In 2014, only April–December statistics were available for the researchers, and 25 hospitals (83%) submitted, with 17 (68%) above 75% of their expected submissions. Overall, 25 facilities’ data were used.

Of the total conditions, the most common conditions treated by physiotherapists within the Gauteng province state health facilities were lower limb fractures (including ankles) (13%) followed by stroke (7.6%) and soft tissue injuries (7.1%) (Table 1). The most common neuro-musculoskeletal conditions were lower limb fractures (27.7%) followed by soft tissue injuries (15.1%) and upper limb fractures (12.9%) (Table 2). The least common neuro-musculoskeletal conditions were facial fractures (0.2%), brachial plexus lesions (0.5%) and traumatic amputations (0.5).

**TABLE 1:** The most common conditions treated by physiotherapists in the Gauteng state hospitals during the period 2012–2014 (*n* = 705 597).

Medical conditions	*n*	%
Lower limb fractures (including ankles)	91 705	13.0
Stroke	53 794	7.6
Soft tissue injuries	49 894	7.1
Upper limb fractures (excluding hands)	42 765	6.1
Arthritic conditions: osteoarthritis	35 156	5.0
Back (discogenic lesions)	34 192	4.8
Intensive care unit patients	32 020	4.5
Cerebral palsy	29 692	4.2
Sputum induction or collection	29 273	4.1
Tuberculosis	23 598	3.3
Developmental delay	20 516	2.9
Hand injuries	20 343	2.9
Head injuries	18 727	2.7
General weakness	14 669	2.1
Burns	14 233	2.0
Neurosurgery	14 217	2.0
Pneumonia	13 062	1.9
Intercostal drains	13 061	1.9
Mastectomy	12 315	1.7
Amputations: vascular	11 923	1.7
Spinal lesions (non-traumatic)	10 309	1.5
Acute lung disease	8764	1.2
Joint replacements	8003	1.1
Peripheral nerve lesions	7524	1.1
Spinal injuries (traumatic)	7180	1.0
Routine pre– or -post-laparatomy	6827	1.0
Other†	81 835	11.6
**Total**	**705 597**	**100.0**

Only conditions > 1% are presented in this table.

† Other: Neonates, Meningitis, High Care or Ortho Acute, Central Nerve Lesions, Cardio-thoracic Surgery or Trauma, Discogenic Lesions, Pelvic fractures, Dislocations, Vertebral fractures (no fall-out), Congenital disorders, Lung Abscess, Rheumatoid arthritis, Caesarean + Gynae, Chronic obstructive pulmonary disease, Spinal surgery, Pre– or -post-natal, Circulatory disorders, Soft tissue reconstructive surgery, Bronchiectasis, Cardiac rehab, Peripheral nerve injuries, Oncology, Asthma, Chronic pain, Chronic lung disease, Extensive general surgery, Amputations: traumatic, Incontinence, Cystic fibrosis, Nephrology, Bell’s palsy, Wound care, Dermatology, Sinusitis, Haemophilia, Urology, Visual impairment, Facial fractures, Stress, Obesity, Laryngectomy, Malaria, Organ transplants.

**TABLE 2:** Neuro-musculoskeletal conditions (including paediatric cases) (*n* = 330 511).

Medical conditions	*n*	%
Lower limb fractures (including ankles)	91 705	27.7
Soft tissue injuries	49 894	15.1
Upper limb fractures (excluding hands)	42 765	12.9
Arthritic conditions: Osteoarthritis	35 156	10.6
Back (discogenic lesions)	34 192	10.3
Hand injuries	20 343	6.2
Amputations: vascular	11 923	3.6
Joint replacements	8003	2.4
Neck (discogenic lesions)	4677	1.4
Pelvic fractures	4202	1.3
Dislocations	4145	1.3
Vertebral fractures (no neurological fall-out)	3891	1.2
Congenital disorders	3770	1.1
Arthritic conditions: Rheumatoid arthritis	3740	1.1
Spinal surgery (including laminectomy)	2977	0.9
Soft tissue reconstructive surgery	2691	0.8
Peripheral nerve injuries (excluding brachial plexus)	2394	0.7
Amputations: traumatic	1742	0.5
Brachial plexus	1724	0.5
Facial fractures (temporomandibular joint/dislocations)	577	0.2
**Total**	**330 511**	**100.0**

The most common cardiopulmonary conditions were tuberculosis (24.9%), followed by pneumonia (13.8%) and patients with intercostal drains (ICDs) (13.8%) (Table 3). The least common cardiopulmonary conditions were organ transplants (0.1%) and laryngectomy (0.1%).

**TABLE 3:** Cardiopulmonary conditions (including paediatric cases) (*n* = 94 895).

Medical conditions	*n*	%
Tuberculosis	23 598	24.9
Pneumonia	13 062	13.8
Intercostal drains	13 061	13.8
Acute lung disease	8764	9.2
Routine pre– or -post-laparatomy	6827	7.2
Cardio-thoracic surgery or trauma	4694	4.9
Lung abscess	3762	4.0
Chronic obstructive pulmonary disease	3300	3.5
Bronchiectasis	2529	2.7
Myocardial infarction and cardiac rehab	2477	2.6
Asthma	2365	2.5
Chronic lung disease	1962	2.1
Extensive general surgery	1852	2.0
Cystic fibrosis	1665	1.8
Head and neck conditions that affect respiration	1408	1.5
Vascular conditions that affect respiration	1186	1.2
Sinusitis	1179	1.2
Urology	982	1.0
Laryngectomy	124	0.1
Organ transplants	98	0.1
**Total**	**94 895**	**100.0**

The most common neurological conditions were stroke (30.9%) followed by cerebral palsy (17%) and developmental delay (11.8%) (Table 4). The least common conditions were general central nerve lesions (excluding spinal cord injury, head injury and stroke) (2.7%) and Bell’s palsy (0.7%).

**TABLE 4:** Neurological conditions (including paediatric cases) (*n* = 174 024).

Medical conditions	*n*	%
Stroke	53 794	30.9
Cerebral palsy	29 692	17.1
Developmental delay	20 516	11.8
Head injuries	18 727	10.8
Neurosurgery	14 217	8.2
Spinal lesions (non-traumatic)	10 309	5.9
Peripheral nerve lesions	7524	4.3
Spinal injuries (traumatic)	7180	4.1
Meningitis	6069	3.5
Central nerve lesions	4711	2.7
Bell’s palsy	1285	0.7
**Total**	**174 024**	**100.0**

Within the non-specified categories, the number of intensive care unit (ICU) patients was the highest (23%), followed by sputum induction (21%) and general weakness (10.6) (Table 5). The least common conditions were malaria (0.1%), obesity (0.1%) and stress (0.3%).

**TABLE 5:** Conditions from non-specified categories (including paediatric cases) (*n* = 138 187).

Medical conditions	*n*	%
Intensive care unit patients	32 020	23.2
Sputum induction or collection	29 273	21.2
General weakness	14 669	10.6
Burns	14 233	10.3
Mastectomy	12 315	8.9
Early intervention or neonates	6128	4.4
High care and acute ortho	4956	3.6
Caesarean + Gynae	3387	2.5
Pre– or -post-natal	2906	2.1
Circulatory disorders (including hypertension)	2705	2.0
Oncology	2382	1.7
Chronic pain	2240	1.6
Diabetes	1813	1.3
Incontinence	1683	1.2
Nephrology (dialysis)	1578	1.1
Wound care	1280	0.9
Dermatology	1241	0.9
Haemophilia	1077	0.8
Other (non-specified conditions)	906	0.7
Visual impairment	744	0.5
Stress	386	0.3
Obesity	148	0.1
Malaria	117	0.1
**Total**	**138 187**	**100.0**

Almost all the conditions listed on the Gauteng physiotherapy patients’ condition statistics are included within the Wits curriculum summary (Appendix 1). Only patients whose primary condition was stress and laryngectomy are not explicitly covered within the Wits curriculum. The most common conditions were emphasised within the Wits curriculum as indicated by the amount of teaching time allocated for these common conditions: fractures, 14.5 (66%) of 22 third-year orthopaedics hours; stroke, 30 (73%) of 41 third-year neurology hours; soft tissue injuries, 18 (38%) of 48 fourth-year neuro-musculoskeletal hours; back lesions, 24 (50%) of 48 fourth-year neuro-musculoskeletal hours; and ICU patients, 30 (79%) of 38 fourth-year cardiopulmonary hours.

### Ethical consideration

Ethical clearance (M140870) was granted by the University of the Witwatersrand Human Research Ethics Committee for research on human subjects and permission to use the physiotherapy patient statistics was granted by the Gauteng health department. The physiotherapists responsible for collation of the physiotherapy patient statistics were contacted to make them aware of the provincial department permission and to request their permission and time to discuss the statistics. The study proposal was presented at the Gauteng Physiotherapy Forum (2014/03/11) to share the plan with Gauteng physiotherapists and to get their advice on possible errors to be aware of when collating the data.

## Discussion

With this paper, we aimed to establish whether the most prevalent medical conditions treated by physiotherapists in Gauteng state health facilities were covered in the Wits physiotherapy curriculum. Eighty-three per cent (25) of the hospitals submitted more than 75% of their monthly statistics over the 2012–2014 data collection period. This information can thus be used to reflect on the type of patients seen by physiotherapists within the Gauteng state hospitals because data are from almost all hospitals within the province.

The Wits physiotherapy curriculum covers all medical conditions treated by physiotherapists within the Gauteng state health facilities and also covers everything within the World Confederation of Physical Therapy (WCPT) minimum training standards (WCPT 2011). Emphasis on the most common conditions within the Wits physiotherapy curriculum is on the most common conditions treated by physiotherapists within the Gauteng state health facilities as indicated by the number of physiotherapy teaching hours allocated for these conditions.

Conditions that are least common within these facilities are also included in the Wits curriculum and this prepares the students to practise in all clinical settings within and outside SA. Examples of these conditions are malaria, which is less common in SA compared to other regions within sub-Saharan Africa (SA Malaria Report 2013). Multiple sclerosis (MS) is also less common in SA but more common in North America and Europe (Atlas of Multiple Sclerosis 2013) and this explains why the number of patients with these conditions is low in the patient statistics for the province. However, these conditions, even though not part of the core curriculum, are covered within the curriculum to prepare students for the global population and the few cases they may encounter within SA. Students can assess patients well and thus if they come across unusual conditions they can cope because they can identify the patient’s problems and related impairments.

Medical conditions seen by physiotherapists within the Gauteng state health facilities are similar to the national disease burden and Wits curriculum. Thus, Wits is teaching what is relevant to our health sector and thus contributes graduates who are useful for the country. Tuberculosis and pneumonia are the most common cardiopulmonary conditions in this study. This may be because of the fact that these conditions are HIV-opportunistic diseases (Global AIDS Response Progress Reporting 2015), and with a high number of people living with HIV in SA (UNAIDS South African 2015), it makes sense that these are among the most common conditions treated by physiotherapists. Bradshaw et al. (2010) also included these conditions in their top six causes of death in SA. Stroke is also one of the most common conditions seen by physiotherapists, and this is expected because of the high rate of hypertension, diabetes, obesity and HIV, which are all risk factors for stroke (Connor et al. 2005; Ortiz et al. 2007). HIV, depression, ischaemic heart diseases and road traffic accidents are the top four 2030 projected causes of burden of disease (Mathers & Loncar 2006). All these conditions or conditions associated with these (e.g. fractures and soft tissue injuries from road traffic accidents, stroke because of heart diseases of HIV) are covered within the Wits physiotherapy curriculum. This addresses what was raised by Stewart et al. (1994) in their study to determine Wits physiotherapy graduates’ contribution to SA’s health needs. They concluded that the graduates over-serviced soft tissue, orthopaedics and respiratory conditions; however, our study shows that these are indeed among the most common conditions. Stewart et al. (1994) also indicated that neurological, spinal cord lesions and cerebral palsy were underserviced. In our study, these conditions are also among the most common conditions treated by physiotherapists and are also covered within the Wits curriculum. Thus, Wits graduates do meet the needs of the current SA health sector.

Fractures, soft tissue injuries and patients with ICDs are also common. This is expected because of the high trauma rate in SA (motor vehicle accidents, stab chest and occupational injuries) (Norman et al. 2007). The high number of ICU patients may be because of the fact that almost all patients in ICU require physiotherapy for chest clearance or mobilisation. There was also a high number of sputum inductions, and this may be because of the fact that tuberculosis is the most common cardiopulmonary condition and some of the patients cannot cough productively; yet, their sputum is required for laboratory tests to confirm diagnosis. Wits students are taught sputum induction and thus will be able to collect sputum whenever indicated.

Developmental delay was also common in this study, and this could be in part because of HIV (Potterton et al. 2010). Children in SA are vulnerable to several other factors causing developmental delay including malnutrition and poverty (Walker et al. 2011). Conditions that appear on the Gauteng patient statistics form that are not explicitly covered within the Wits curriculum are stress and laryngectomy. These are covered indirectly because students learn skills to manage symptoms of patients who may present with stress such as pain, muscle spasm, poor endurance and somatoform disorders and also teach patients exercises and personal management to prevent or manage the stress-related impairments or problems (Kaur, Masaunz & Bhatia 2013). This is covered during the community physiotherapy block; however, after graduating, students have opportunities to do short courses if they have to focus on stress management.

Laryngectomy is not covered, but students do cover respiratory techniques including suctioning that are required for the management of patients post-laryngectomy. Laryngectomy is usually performed for patients with cancer of the larynx, and in oncology, they cover the systemic effects of cancer and the role of physiotherapy in treating these. Patients with laryngectomy may have a tracheostomy in place and tracheostomy management and care is also part of the Wits ICU curriculum. The focus in the Wits curriculum is primarily on lung, breast and cervical cancer as these are the most common types of cancers. However, graduates will be able to cope with any cancer condition that requires cardiopulmonary, neurological or musculoskeletal clinical skills because they can assess and identify problems and possible causes.

The results of this study indicate that all conditions treated by physiotherapists within the Gauteng province are covered by the Wits physiotherapy curriculum. The curriculum actually covers more conditions than those that appear on the Gauteng physiotherapy patient statistics form, and this is to ensure that students are trained to work in any clinical facility throughout the world even though the core business is the SA context. Thus, the Wits physiotherapy curriculum accommodates the necessary generic skills to ensure mobility and enhance quality of life.

All medical conditions identified as the most common by Bradshaw et al. (2010) are covered within both the Wits curriculum and Gauteng statistics form. The conditions are also in line with what the WCPT considers within the minimum physiotherapy training standards as indicated in this portion of the WCPT (2011) document:

The physical therapist professional curriculum includes content and learning experiences in the clinical sciences (e.g. content about the cardiovascular, pulmonary, endocrine, metabolic, gastrointestinal, genitourinary, integumentary (skin), musculoskeletal and neuromuscular systems and the medical and surgical conditions frequently seen by physical therapists). (p. 11)

## Limitations of the study

This was a record review, and thus, the authors did not have control over the development, submission and collation of provincial statistics.

## Conclusion

The Wits physiotherapy curriculum covers all medical conditions treated by physiotherapists within the Gauteng state health facilities. Conditions that are least common within these facilities are also included in the Wits curriculum, and this prepares the students to practise in other worldwide clinical settings where such conditions are common. The conditions are also in line with what the WCPT considers to be the minimum physiotherapy training standards. Medical conditions treated by physiotherapists within the Gauteng state health facilities are within the Wits curriculum and on the list of the national and international disease burden.

## APPENDIX 1

**APPENDIX 1:** Summary of conditions covered within the University of the Witwatersrand Physiotherapy curriculum.

Neuro-musculoskeletal conditions (including paediatric cases)		Cardiopulmonary conditions (including paediatric cases)	Neurological conditions (including paediatric cases)
**Conditions of the spine** Muscle sprain, spasm, injury guarding Discogenic – bulge or protrusion Degenerative arthritis Zygapophyseal joints (facet) – capsular strain, tear, cartilage damage, degenerative arthritis Fractures of the vertebral body and spinous process Fascia, ligament Mechanical and inflammatory low-back pain Inflammatory syndromes: Ankylosing spondylitis; psoriatic, reactive and rheumatoid arthritis; polymyalgia rheumatic Spinal hypomobility, hypermobility or instability Hypermobility syndromes: Marfan syndrome; Ehlers–Danlos syndrome Nerve root irritation, compression, stenosis Spondylolysis, spondylolisthesis Spondylosis/osteoarthritis Lumbar radiculopathy Metabolic syndromes: osteoporosis; osteomalacia; Paget’s disease Infections: TB-spine; osteomyelitis Neoplasms: benign or malignant Visceral pathology: Abdominal aortic aneurysm; endometriosis; prostatitis Fibromyalgia, myofascial pain syndrome Piriformis syndrome, postural pain syndrome Coccydinia, sacro-iliac joint dysfunction Thoracic outlet syndrome, T4 syndrome Visceral pain of thoracic origin Scheuermann’s disease (vertebral osteochondrosis), Tietze’s syndrome Whiplash-associated disorders Discogenic wry neck, acute locked joint Conditions that may mimic Cx, upper Tx or shoulder girdle symptoms: Malignant lymphadenopathy; Pancoast’s tumour; Vertebral artery syndrome; subarachnoid haemorrhage; coronary artery disease, polymyalgia rheumatic; angina; carcinoma of the bronchus; diaphragmatic pleurisy; secondary malignant deposits at the scapula; herpes zoster; glandular fever Headache: cervicogenic; migraine without aura; tension type Vertebro artery insufficiency Temporomandibular joint dysfunction	**Neurodynamics** Intraneural pathology: neuroma; demyelination; scarred epineurium; arachnoiditis; irritated dura mater Extraneural pathology: adhesion of dura to posterior longitudinal ligament; swelling of bone and muscle adjacent to a nerve trunk; narrow spinal canal Injury as result of vascular factor; mechanical factor; axoplasmic flow, Neural fibrosis Double crush syndrome Neuropathy, sympathetic nervous system involvement **Peripheral nerves** Neurapraxia, axonotmesis Neurotmesis, Erb–Duchenne paralysis or Erb’s palsy (upper trunk injury), Klumpke’s paralysis (lower trunk injury), Saturday night palsy (lower trunk injury) Horner’s syndrome, hand of benediction, Carpal tunnel syndrome, nerve injuries: median; radial; ulnar; posterior tibial; common peroneal nerve, sciatic nerve palsy, drop foot Reflex sympathetic dystrophy – Sudeck’s atrophy **Shoulder complex** Instability, Scapula dyskinesis Subacromial impingement (primary impingement) Secondary impingement Supraspinatus tendonitis Rotator cuff tear, adhesive capsulitis, acute calcific tendonitis Biceps tendonitis, cervical radiculitis, Pancoast’s tumour Neoplasm **Orthopaedics** Fractures and dislocations Hand injuries, shoulder injuries Hip and thigh injuries, elbow injuries, knee injuries Leg injuries or pain Foot and ankle injuries Osteoarthritis and rheumatoid arthritis, Amputations Fractures or dislocation of the appendicular skeleton Arthroplasty Diseases of bone and joint: Osteomyelitis, septic arthritis, avascular necrosis, gout, TB Spinal surgery	Upper respiratory infections Complications of bed rest Restrictive lung diseases: Pneumonia (Pneumocystis jiroveci pneumonia ventilator associated pneumonia other bacterial pneumonias), TB, lung abscess, interstitial lung diseases, acute respiratory distress syndrome Asthma Bronchiectasis, atelectasis Chronic bronchitis and emphysema Chronic lung diseases Burns and inhalation injuries Diabetes mellitus, obesity, metabolic syndrome and hypertension HIV Oncology: lung cancer, breast cancer, cervical cancer Tracheostomy management and care Cardiac valve diseases and surgery, myocardial infarction, angina pectoris, coronary artery bypass graft surgery Cardiac arrhythmias and cardiac failure Chest trauma: rib fractures, sternal fractures, pneumothorax, haemothorax, pleural effusions, empyema Lower respiratory tract surgery Pre- and post-operative management of surgical patients Respiratory failure, shock states Acute renal failure Acute brain injuries Acute spinal cord injuries Polytrauma patient in ICU Guillain–Barre patient in ICU Cystic fibrosis Sputum induction	Stroke Traumatic brain injury Spinal cord injury Parkinson’s disease Motor neuron disease Multiple sclerosis Guillain–Barre syndrome Prematurity Cerebral palsy: ataxia, hypertonicity, hypotonicity, athetosis Spina bifida Spinal muscular atrophy Duchenne muscular dystrophy Developmental coordination disorder HIV TB Pneumonia Cystic fibrosis Bronchiectasis Congenital cardiac lesions Developmental delay because of a number of factors Haemophilia Head injury Meningitis Burns Hydrocephalus Fractures Scoliosis Post-operative complicated selective orthopaedic surgery, for example, single-event multilevel surgery (SEMLS) Sports injuries in kids Myasthenia gravis Epilepsy, neurosurgery Autism Normal development Common neonatal disorders **Non-specified categories** Geriatrics Women’s health Occupational health Metabolic disorders Tumours and disorders of growth Chronic kidney disease Chronic liver disease and hepatic failure Parathyroid disorders Systemic lupus erythematosus General urology Primary skin diseases

ICU, intensive care unit; TB, tuberculosis.
